# An absurdist ethics of AI: applying Camus’ concepts of rebellion and dignity to the challenges posed by disruptive technoscience

**DOI:** 10.1007/s00146-025-02482-9

**Published:** 2025-07-15

**Authors:** Marlon Valentijn Kruizinga, Hub Zwart, Valerie Frissen

**Affiliations:** 1 Erasmus University Rotterdam, Rotterdam, Netherlands; 2 Leiden University, Leiden, Netherlands; 3 Leiden University, Leiden, Netherlands; 4 SIDN Fonds, Arnhem, Netherlands

**Keywords:** Artificial intelligence, AI ethics, Ethical dilemma, Albert Camus, Absurdism, Technological mediation

## Abstract

This article proposes a new, Camusian approach to analyzing and navigating ethical dilemmas in relation to Artificial Intelligence (AI) and, by extension, to other disruptive technoscience. The article takes as its point of departure the *Unified Framework of Five Principles for AI in Society,* as advanced by Floridi and Cowls (2021), which offers a comprehensive and cohesive framework of the many abstract values and principles brought up in AI ethics discourse. Using a case-study approach, which focuses on the principle of accountability in AI, we demonstrate that, even following an exhaustive systematization of abstract principles, ethical dilemmas still arise whenever we consider applications of the technology in concrete situations. Furthermore, because of the way technology mediates our ethical judgement, a deeper ethical dilemma arises; we can either judge technologies like AI prematurely, without knowing their impact, or after they have been able to bias our norms and intuitions for ethical deliberation. This article then argues that the vulnerability of our ethical judgement to continuous doubt, which is exposed by AI as a landmark case of disruptive technology, can be addressed by integrating Albert Camus’ philosophy of absurdity, rebellion and dignity. Through Camus, we can contextualize our experience of ethical doubt in relation to AI as existentially absurd, while also navigating normative change more confidently with meta-level principles for ethical deliberation itself. The article concludes that, while ethical dilemmas and doubts will persist, this Camusian approach will make us more responsible in undertaking the continuous adaptation and concretizing of our ethical frameworks.

## Introduction

Artificial Intelligence (AI) is an emerging, pervasive, and disruptive technology that presents a plethora of societal contexts with potential new practical possibilities. In the context of this article, we will follow the definition of AI offered by Floridi and Cowls, who define it as “a growing resource of interactive, autonomous, and often self-learning agency […], that can deal with tasks that would otherwise require human intelligence and intervention to be performed successfully” (2021, p. 7). This means that we must consider AI as not only being able to change the ways and degrees in which people can affect each other (as is the case with any tool), but also as something which, in itself, may affect us on a more systemic level. The new capacities of AI, as well as the data-driven form of its creation and operation, have led to considerable ethical debate, as academics, legal practitioners, and politicians alike attempt to keep pace with AI’s development and dispersion into society.

This article contributes to the academic ethical discourse on AI, using a case-study approach to indicate how AI is deeply entangled with our societal and political contexts. Notably, we will discuss the ways in which AI (and other socially disruptive technoscientific innovations) can throw our conventional ethical standards and assumptions into disarray. We also explore possibilities to restructure our ethical concepts around the new technological realities with which we are faced. We will do so through a somewhat unusual philosophical lens: that of Albert Camus’ absurdist and rebellious moral philosophy, as outlined in *The Myth of Sisyphus* and *The Rebel* (2013a; 2013b).^1^

To begin, we will introduce one of the leading perspectives on the ethics of AI, namely Floridi and Cowls’ *Unified Framework of Five Principles for AI in Society*, which systematizes the free-floating ethical principles that have emerged in response to society’s ongoing experiences and concerns with regard to AI (2021). Subsequently, we will demonstrate that the system of principles they introduce, while comprehensive and cohesive, still leaves room for ethical dilemmas to emerge when the framework is applied in more concrete contexts of AI use. We shall analyze one example of such ethical dilemmas, namely the dilemma of accountability in AI. Using this prominent ethical aspect of AI, we show that an ethical dilemma arises from (likely intentional) ambiguities in the terms and concepts that make up Floridi and Cowls’ framework of five principles.

Additionally, however, this article will examine an attempt to address the dilemma of accountability in AI. This examination will then illustrate a deeper, underlying dilemma in the ethics of technology created by the technological mediation of our societal contexts and normative judgements (Verbeek and Crease 2005). This mediation, we argue, fundamentally problematizes our attempts to make the sorts of abstract ethical AI principles advanced by Floridi and Cowls more concrete. It does so by calling into question the reliability of our collective ethical judgement. Through technological mediation, we are in danger of only judging innovations ‘on their own terms’, or otherwise of judging them prematurely (Kudina and Verbeek 2019). AI here functions as a landmark case, since it confronts us with the broader issue of technological mediation and ethical dilemmas in a particularly intense and widespread manner. The problem thus uncovered cuts deep: it suggests that none of our conventional moral principles are truly trustworthy, as they are constantly reshaped and mediated by material, technological change. Far from perfectly rational, we must understand our normative judgements to be marred at all times either by ignorance or bias (or, more likely, by a combination of the two).

After this preliminary analysis, we will argue that the solution to this deeper dilemma, of our judgement being either premature or technologically mediated, can be found in Camus’ philosophical work on ‘rebellion’ and ‘the absurd’. First, Camus’ thinking helps us connect our position of ethical doubt in relation to technologies such as AI with the more general existential experience of absurdity, which Camus analyzes in *The Myth of Sisyphus* (2013a). In both cases the human need for understanding and normative guidance is frustrated by the fundamental indeterminacy of our situation (as new developments simultaneously motivate and problematize normative adaptations). Second, it allows us to see that the ethical frustration experienced in both cases of absurdity lead to ‘rebellious struggle’: the rejection of previously established, unsatisfying normative orders and the attempt to build new ones, which Camus examines in *The Rebel* (2013b).

Finally, this article argues that the values and concepts considered by Camus to be internal to rebellious struggle offer a touch stone by which a change in normative order is guided through the human condition of persistent (self-)doubt. To be precise, the Camusian value of ‘human dignity’, as well as a logic of self-consistency seen as internal to rebellion, must together form the basis for ‘responsibly’ adapting our ethical frameworks in a given societal context. They would allow us to concretize abstract principles (such as those put forward by Floridi and Cowls for AI) for a particular context, without relying solely on that context’s conventionalized and technologically mediated ethical assumptions. To formalize these Camusian insights, we distill from the aforementioned values and concepts three meta-level normative principles, which are intended to guide further ethical deliberation and (re)consideration within particular societal contexts where AI (and other technoscientific innovations) may cause dilemmas and doubts. What is thus offered is not a cure for ethical doubt or dilemma, as these are shown by both Camus and the case of AI ethics to be permanent fixtures of a human condition that is honest with itself. Rather, what is offered by our Camusian meta-level principles is guidance that can make this absurd condition navigable, making it at least bearable for those who doubt their judgement but simultaneously refuse to give up on shaping the future responsibly.

### Ethically adapting to AI with a system of principles

The idea that AI, as a new technology, forces us to rethink and adapt our normative frameworks is certainly not new. The introduction of the *AI Act* by the European Parliament represents a clear recognition that previous legal frameworks were not sufficient to “ensure that Europeans can benefit from new technologies developed and functioning according to Union values, fundamental rights and principles” (The Act | The Artificial Intelligence Act 2023, p. 1). In addition to proposing new legislation, this particular document recognizes the need to clarify some of the conceptual understandings internal to our legal system by establishing “a technology-neutral, uniform definition for AI that could be applied to future AI systems” (EU AI Act 2023).

It is clear that ethics research also has not sat still with regard to these issues. As previously mentioned, we shall, in this article, operate on an understanding of AI as formulated by Floridi and Cowls: i.e. “a growing resource of interactive, autonomous, and often self-learning agency […], that can deal with tasks that would otherwise require human intelligence and intervention to be performed successfully” (2021, p. 7). Beyond merely this definition, the work of Luciano Floridi in particular has, in recent years, served to practically define the ethical debate around Artificial Intelligence (especially in Europe). From 2018 onwards, Floridi chaired the AI4People initiative, launched by the European Parliament, which “combines efforts of a scientific committee of international experts and a forum of stakeholders, in consultation with the High-Level Expert Group on Artificial Intelligence of the European Commission, to propose a series of concrete and actionable recommendations for the ethical and socially preferable development of AI” (Taddeo and Floridi 2021, p. 94).

In this article, then, we take into account the highly significant contribution that Floridi, as well as other researchers, have already made to the field of ethics of AI. This work, too, highlights the need presented by AI to reconsider our specifically ethical frameworks in light of the societal impact of the new technology. In their *A Unified Framework of Five Principles for AI in Society*, Floridi and Cowls note the following:

The increasing demand for reflection and clear policies on the impact of AI on society has yielded a glut of initiatives. Each additional initiative yields a supplementary statement of principles, values, or tenets to guide the development and adoption of AI. The risk is unnecessary repetition and overlap, if the various sets of principles are similar, or confusion and ambiguity, if they differ. (2021, p. 8)

Floridi and Cowls go on to present five principles which they believe present “a coherent and sufficiently comprehensive overview of the central ethical principles for AI”, which could then “serve as the architecture within which laws, rules, technical standards, and best practices are developed for specific sectors, industries, and jurisdictions” (2021, p. 14). Their proposed five principles for AI, partially modelled after the bioethical principles of Beauchamp and Childress, are set forth below:

- *Beneficence*, meaning that AI should be made and used for the betterment of people and the planet.
- *Non-maleficence*, meaning that neither the functions nor the (over)use of AI should be allowed to do harm to people.
- *Autonomy*, which supplements the previous principles by insisting that AI must benefit and not harm humans’ ability to have executive control over their own lives and objectives.
- *Justice*, which puts the emphasis on equity and non-discrimination in the uses and benefits of AI.
- *Explicability*, which supplements all previous requirements by stating that particular and appropriate persons must be held accountable for the design and implementation of AI, as well as particular persons being responsible for the provision of (intelligible) information to the public to make external scrutiny possible (Floridi and Cowls 2021, p. 10–12).

This set of principles already does much to distill and systematize the many values and principles which have begun to float around in the public and academic discourse in relation to AI. Therefore, it is important to note that our aim here is not to undermine these principles, as much as it is to help build on the groundwork they have laid. In particular, this article claims that when it comes to filling in these principles in more concrete contexts, there will arise additional ethical dilemmas and open conceptual questions, which can be aided in their answering by a Camusian approach to ethics. The bioethical principles of Beauchamp and Childress—which include all the same basic concepts as those of Floridi and Cowls, except for Explicability—were notably formulated to function in a situation where fundamental disagreement concerning the foundation of morality, or concerning concrete implications of abstract principles, was an accepted possibility (Beauchamp and Childress 2001, p. 22). This further indicates that these principles for AI are, at their origin, not intended to be directly applied to more concrete practical situations, without any further translation or interpretation. On the contrary, the principles were formulated with the expectation that ethical disagreements within their contexts of application would still follow and may not be entirely undesirable.

**Fig. 1:**
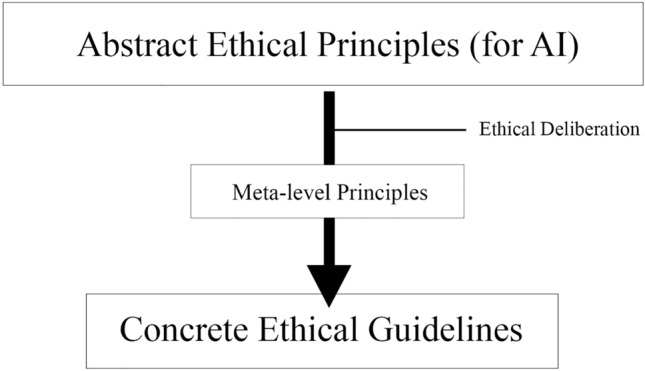
Meta-level principles are placed within the deliberative process of concretizing Abstract Ethical Principles into particular decisions and guidelines

The Camusian approach introduced in this article will, therefore, provide no mere supplementary principles for ethical AI, but instead develops a set of meta-level principles which can be used to guide our deliberation on ethical dilemmas and reconsiderations in concrete situations (Fig. 1). These are principles for deliberation on ethics, not principles with their own ethical demands outside the deliberative context. The meta-level principles are intended to be of use precisely in situations where the exact meaning and weight of abstract ethical values and principles have been cast into doubt, prompting deliberation on- and reconsideration of the ethical framework of a particular (societal) context. The reason these meta-level principles are here introduced in the context of AI is that, as a socially disruptive technology, it confronts us with especially acute cases of the ethical doubt described above. In these cases, namely, multiple ethical dilemmas arise at once which each, respectively, confronts us with the insufficiency of our current, conventional understandings of particular values and principles, causing us to challenge and doubt these understandings. In addition, confronting disruptive technologies should also cause us to doubt our ability to make responsible, rationally satisfying decisions about how to reconsider our meanings and change our ethical frameworks.

To illustrate this multifariously ethically disruptive effect of AI, we will in the next sections review one major ethical dilemma caused by the introduction of the technology in particular societal contexts: the dilemma of accountability in AI. This dilemma relates directly to the built-in ambiguities in the ethical principles for AI introduced by Floridi and Cowls, namely in the principle of explicability, which emphasizes the necessity of additional work to concretize these principles in particular situations. We will then examine a proposed solution to the dilemma of accountability in AI, to highlight a deeper dilemma of technological mediation, which arises whenever we try to reconsider our ethical frameworks in response to a novel technological tool. This ‘surface dilemma’ and ‘deeper dilemma’ are subsequently combined to show why the emergence and adoption of AI requires not only ethical reconsideration/deliberation, but, in addition, meta-level principles for guiding said reconsideration responsibly. These meta-level principles are, of course, not intended solely for ethical deliberation on AI. However, AI serves as both a highly relevant use-case, and a strong illustrative example of technology-induced ethical doubt involving both types of dilemmas.

### The dilemma of accountability

A central example of the kinds of conceptual ethical reconsiderations which AI (and other algorithms) motivates us towards concerns the concept of ‘accountability’. The relevance of ‘accountability’ as a concept for the context of AI is only underlined by the fact that Floridi and Cowls included it in their principle of explicability, which is the one principle they explicitly added to the ethical principles they adapted from Beauchamp and Childress’ principles for bioethics. It seems, then, that explicability (and thereby accountability) is a principle that is especially relevant to- and affected by AI, as opposed to previous technoscientific innovations. The reason for this, as we shall see, is that unlike most other technologies AI appears to introduce a sort of agency of its own into the more familiar human–machine pairing. This factor is clearly addressed by the principle of explicability, as it states that particular and appropriate persons must be held accountable for the design and implementation of AI (Floridi and Cowls 2021, p. 12). However, the ambiguity here, which leads to one—if not several dilemmas, is who these ‘appropriate persons’ are, and whether AI does not problematize finding them.

Mecacci and Santoni de Sio, in their recent article, recognized at least 4 different types of responsibility/accountability gaps created by the introduction of AI: gaps in culpability (legal as well as moral), in moral and public accountability, and in active responsibility (2021, p. 1059). Here, they highlight how the presence of AI in a system, be that a government system or another human-operated technological tool such as a car, can make it difficult to assign blame to the proper parties (or, at times, to any human parties at all) when the system in question causes harm. This culpability gap arises because the AI takes up a normally human place in the network of agencies that produces the choices and actions we seek culpability for, further intensifying the classical ‘problem of many hands’ within agent networks (Miceli and Bovens 1999, p. 846), and because the opacity of the decision-making process within the AI can make it hard for AI-users to fulfil the conditions of knowledge and intent necessary for classical culpability (Santoni de Sio and Mecacci 2021, p. 1062–1063).

Furthermore, Mecacci and Santoni de Sio show that this same internal opacity of AI, its contribution to agent networks’ complexity, and the entanglement of the technical explanations which do exist in corporate intellectual property, problematize normal standards of moral- and public account-giving (2021, p. 1064). A particular AI-user may not understand the AI they use well enough, or only have such an incremental role in its use, that they are unable to clearly explain why its use creates certain outcomes or leads to certain choices.

Finally, Santoni de Sio and Mecacci argue that our current understandings of the social roles and responsibilities of such parties as developers, engineers, public officials, and private tech-users are not well-suited to clarifying and motivating the active, forward-looking responsibility for designing and operating AI in a safe and publicly beneficial way. The designers may see their role as merely optimizing the AI’s effectiveness, thinking that the officials who use or license it are responsible for judging its safety, while officials and citizens may see the designers as the experts with the best chance at safeguarding against negative outcomes of the AI (Santoni de Sio and Mecacci 2021, p. 1067). The designer may also not understand the precise public purpose for which their AI will be used, and public officials may not understand the technical aspect of the AI well enough to fully judge the safety of its deployment (Santoni de Sio and Mecacci 2021, p. 1068). In short, the way in which public usage of AI creates decision chains between public, private and civil entities, and between different areas of expertise which may be rather ignorant of each other, problematizes each of these parties understanding what their responsibilities are, let alone feeling motivated and able to discharge them.

### A solution for AI accountability

Santoni de Sio and Mecacci, recognizing that these accountability gaps are created in part by an inadequacy in our current moral and legal frameworks of accountability, then attempt to supplement these frameworks with a “philosophical account of MHC [Meaningful Human Control]” to create “a unified conceptual framework which also provides some principles to practically (re)configure AI to minimize possible responsibility gaps” (2021, p. 1075). According to them, to be able to hold each other accountable for the design, development and deployment of AI to a satisfactory degree, we must first adapt our understanding of what ‘meaningful human control’ means. The control in question, they argue, is meaningful not (only) when the behavior of an AI-based system was clearly linked to the behavior of a human, but more generally when a human’s reasons/intentions and capacities can be causally connected with the behavior/outcomes of the system (Santoni de Sio and Mecacci 2021, p. 1077). Notably, they also argue that the ‘system’ in question is not just the AI, but also the whole social system (such as the pipeline from developer to government usage) into which the AI is integrated. This entire ‘socio-technical system’ should then allow us to understand how each member’s reasons and capacities figure into the system’s operation. For instance, the profit incentive plus tech-expertise of an AI developer and the efficiency requirements plus legal/administrative powers of a public entity are all forms of meaningful human control, which cause a socio-technical system to produce particular outcomes for people in society, not merely the few engineers who developed an AI and the ground-level public workers who end up using it to help make their decisions. The ability to pin down these kinds of control through reasons and capacities is called the ‘tracking condition’ (Santoni de Sio and Mecacci 2021, p. 1077–78).

In addition, there is the condition that particular reasons and capacities which made a meaningful difference in the system’s behavior are always traceable to particular, discrete individuals (Santoni de Sio and Mecacci 2021, p. 1078–79). Together these ‘tracking and tracing’ conditions would supposedly enable us to assign blame to clear particular parties in case of harm, to allow each person in the system to explain their own particular role or the role of their reasons and capacities, and also to motivate each person to behave responsibly and accountably within their particular role.

### A deeper, contextual problem

The reconsideration of ‘meaningful human control’ by Santoni de Sio and Mecacci certainly goes a long way towards adapting our moral and legal understandings of accountability and responsibility to the realities imposed by AI. There is little doubt that their work is the exact kind of further ethical consideration and framework-building which Floridi and Cowls intended to have built on top of their abstract ethical principles. However, the normative framework introduced by Santoni de Sio and Mecacci still holds plenty of ambiguities. For instance, it is said that the ‘tracking and tracing requirements’ requirements can help “to identify the policy, legal, technical, and organizational interventions needed to ensure that the [socio-technical] system is sufficiently responsive to the right reasons of the relevant agents” (2021, p. 1078). Yet, what ‘reasons’ are the ‘right’ ones is here left entirely open. As Theodore Lechterman points out in his chapter *The Concept of Accountability in AI Ethics and Governance*: “People disagree profoundly about the general ethical principles with which AI should comply” (2022, p. 9). Thus, still, we are far from clear on when an AI has produced an unacceptable result for which someone must be culpable, and we do not really know what would count as an acceptable excuse/reason: “When we hold AI accountable, should we take primary concern with intentions, actions, or results” (Lechterman 2022, p. 10)?

Furthermore, the ‘relevant agents’ within MHC are established to be those whose reasons and capacities connect causally to the behavior of the system, but what constitutes sufficient ‘causal connection’ to merit evaluation of these reasons and capacities seems to be left to further deliberation, or to some sort of ‘common sense’. What emerges in both cases is a persisting reliance on societal ethical deliberations and conventionalized understandings to ‘fill in the blanks’.

One may argue that this is not a bug, but a feature, since the principles of Floridi and Cowls presumed persistent ethical disagreement, and settling every concrete ethical issue cannot- and should not be the purview of academics. Both of these points are correct, and it should once again be emphasized that frameworks like those of Floridi and Cowls, as well as Mecacci and Santoni de Sio, are not invalidated by the ambiguity built into them. However, our aim here is to point out that persisting ambiguity in normative frameworks necessitates the reliance on conventionalized, contextual understandings of normative values and concepts. In other words, when trying to specify the content of those frameworks for a particular context, we will of necessity be subject to the conceptual and evaluative understandings of that particular context. And, as we will see next, this may become a problem when the new (technological) development(s) which we aim to judge already influence the normative context within which we judge them.

Keeping with the case-study of accountability in AI, and Mecacci and Santoni de Sio’s MHC framework, what if our conventionalized normative understandings are developing in such a way that access to the reasons and capacities of certain parties becomes increasingly limited? Santoni de Sio and Mecacci themselves mention earlier on that:

[…] the whole process of technology development and production is arguably pervaded by an increasing pressure towards deploying proprietary technologies that, even when working through mechanisms accessible to their developers and programmers, are designed to be inaccessible to public scrutiny and the users themselves. (2021, p. 1064)

This factor of proprietary technologies could cause a deep shift in the normative context, where access to particular explanations becomes less and less normalized. The legal requirement of tracing particular reasons behind an AI-powered welfare system’s choices back to particular agents may be hindered by the established legal understanding that the AI’s internal code, or its training data, are the protected intellectual property of a company. The ethical demand for explaining the aforementioned reasons behind a decision may be stifled in public discourse by the idea that secrecy on code and training data give an AI company a competitive advantage to which it has a right. The most salient point here is that AI and the practices of innovation surrounding it have the potential to affect the very standards which judge them, and which are expected by many to account for their potential harms. This problem is not exclusive to AI either. It is an instance of a more general dilemma in ethical judgement caused by technological mediation, which is the subject of the following section.

### Ethical dilemmas and technological mediation

Technology, even when it is not a ‘resource of intelligent agency’, meaning it does not actively take over any of our agency-expressing activities, can still change those activities and their seeming moral qualities through mediation. Peter-Paul Verbeek, from a postphenomenological perspective, describes this as the ‘co-constitutive’ relationship between humans, technologies, and their environment, wherein not just the human constructs the machine but each reconstitutes the other (Verbeek and Crease 2005). A car may, on the one hand, enhance our agency to do more than our bodies normally allow (Danaher 2019, p. 133). Yet, it may also change the way we view the world around us, in both a direct and a more abstractly ethical sense (Boer and Kudina 2018, p. 2). Normally, when I do not notice someone’s presence nearby me (in time) and thereby harm them, such as by pushing them over, I may be blamed quite exclusively for my inattention. But if I am in a car, on the highway, and I hit a person who runs across the road, I may be blamed to a far lesser degree. It may be argued that the car’s characteristic high speed and more limited peripheral vision made it hard for me to see the person. Or it may be said that a highway is intended for cars, and the person who was hit should have known to avoid the entire area. Note that all these arguments have to do with how the technological mediation of my (movement) agency by the car, and the infrastructural context built around it, carry with them their own (ethical) standards. This mediating quality of technology, even apart from the way AI can take over human agency-expressing activities, moves us to question, not just what the ethical thing to do *is*, but also what we want it *to be*. After all, if humans and technology co-constitute each other—including the constitution of ethical standards by technologies—then in designing and implementing technologies we are also, in turn, deciding the trajectory of our future ethical standards.

These decisions only become more pressing when technologies like AI threaten to change our practical, normative contexts on a deeper level than we are used to, such as how they could give less sense to taking on- or ascribing accountability at all. This scenario differs from a classical accountability gap in that, when a gap arises, we know that someone ought to be accountable. We just cannot find anyone in particular to blame, punish, or ask for explanations. In a case such as is described by John Danaher in *The Rise of the Robots and the Crisis of Moral Patiency*, the issue is rather that robots and AI may take over our cognitive and practical tasks to such a degree that we come to view ourselves, and each other, less and less as practical, *moral* reasoners, and more and more as the passive subjects of technological beneficence (2019, p. 135). Danaher argues that the capacities necessary for moral agency, the “ability to regulate one’s actions in light of moral rules and values”, can be “impaired or limited in certain environments or at certain stages of maturation (2019, p. 132).” The foremost way for this to happen, besides for instance having a severe (mental) disability, is for a person to be demotivated in exercising their agency-expressing activities, i.e. in performing those actions and reasonings which exercise the capacity for (moral) understanding and self-guidance by understanding (Danaher 2019, p. 133–134). And, as it happens, it is precisely these activities that robotics and AI aim to take over in many areas of life:

If a machine-learning algorithm tells me that the right thing to do is to deny social welfare to someone on the grounds that they could be committing fraud, then it is true to say that I express some minimal form of agency by following through on that recommendation. But the agency in question really is minimal and not strongly moral. If I do not understand the rationale or basis on which the recommendation was made, and if I am not inclined or able to second-guess the algorithm’s suggestion, I am little more than a ‘rubber stamp’. (Danaher 2019, p. 135)

This gives rise to what Danaher calls the ‘Crisis of Moral Patiency’, and the result is less a gap in accountability and more the total breakdown of responsibility and accountability practices. In such a scenario, the very practice of ‘holding accountable’ will come to make less sense to us, as we defer to automation for more and more vital, morally charged actions and choices.

From a postphenomenologist technological mediation perspective, we may say that the transformation of our self-perception as agents is already underway, as we are already surrounded by (AI-based) technologies that take over cognitive and practical tasks, such as self-driving cars and decision-support systems. Since our context of living, and thereby our normative frameworks, are already partly changed, how do we judge in good confidence which degree of (for instance) lost agency and individual accountability is too much?

It is this far-reaching problem of technological mediation which, as this article argues, implies that we cannot rely purely on existing moral conventions—on so-called ‘common sense arguments’—to adapt our moral frameworks for dealing with novel technoscientific innovations and the ethical dilemmas they bring up. Floridi and Cowls’ comprehensive and cohesive system of five ethical principles notwithstanding, when we come down to defining and concretizing these principles for particular contexts of AI use, trying to solve a dilemma or fill an accountability gap, we are inevitably still faced with a deeper ethical dilemma of technological mediation and doubtful ethical judgements. This dilemma can, in line with Verbeek, also be described as an ethical variant of the Collingridge Dilemma:

[W]hen we develop technologies on the basis of specific value frameworks, we do not know their social implications yet, but once we know these implications, the technologies might have already changed the value frameworks to evaluate these implications. (Kudina and Verbeek 2019, p. 293)

On the one hand, we would like to adapt technologies like AI to the proper societal values already in the design phase, before they are properly created or introduced. Yet, these technologies, once created and introduced, might challenge our established value frameworks in unforeseen ways. On the other hand, these technologies, once introduced, will not only challenge but also mediate our ethical judgements. Thus, once we feel the need to adapt our value frameworks to a new technology like AI, it has almost always already influenced our judgement in a way that calls into question any subsequent change in terms of bias.

### A Camusian approach to ethical dilemmas

The ‘deeper dilemma’ in ethics of technology described in the previous sections should make us uncomfortable with handling the more ‘surface level dilemmas’, such as those of accountability in AI. Being aware of- and yet unable to mitigate technologically mediated bias, we are at best irresponsible if we approach the concretization of abstract ethical principles for practical purposes without a guiding structure based on a deep theoretical understanding of the ‘mediation dilemma’ itself. We, therefore, advocate for a Camusian approach to ethics to guide the normative re-evaluations necessary to concretize abstract ethical principles, such as those for AI.

To begin, we will introduce the philosophy of Albert Camus in *The Myth of Sisyphus*, showing how his work recontextualizes our encounters with disruptive technologies like AI as being ‘absurd’ (2013a). This experience of absurdity is then shown to undermine, in a way that is by now familiar, our conventional ethical judgements and assumptions. As this experience simultaneously motivates and problematizes the struggle to adapt our normative status quo, we shall then see how Camus, in his book-length essay *The Rebel*, introduces the concepts of ‘rebellion’ and ‘dignity’ to guide the struggle for normative change.

### AI and the absurd

The philosophy of Camus operates on at least one fundamental characteristic of human existence, namely its absurdity. ‘The absurd’, in the context of Camus’ writings, describes a relationship between the human being and the world, wherein the human longs for rational sense-making, while the world (or nature, or reality) seems to resist being exhaustively and coherently explained: “The absurd is born of the confrontation between the human need and the unreasonable silence of the world” (2013a, p. 22). For our purposes, what needs to be understood is that, for Camus, only exhaustive explanation and justification—involving some fundamental truth(s) accounting for all others without having to be accounted for itself—can lead to certainty in knowing (Hochberg 1965, p. 89). Yet neither philosophy nor science seem able to give such an account of our- and the world’s existence, being eventually reduced to either assumptions, abstract ideas, or scientific models of entities and processes which we cannot truly perceive (Camus 2013a, p. 16–17). Thus, all our supposed knowledge and beliefs retain an irreducible degree of uncertainty, which leaves the rational human drive to understand in a state of frustration. Intellectually speaking, our uncertainty is punctuated whenever our scientific theories shift over time, which frustrates the drive to fully explain the world. Meanwhile, as rational agents, we want to act on what we know to be true and whatever follows reasonably from it—a sort of pre-moral normative aim which Camus refers to as ‘decency’—but, since we cannot establish certainty in knowing, we also cannot act with full confidence (2013a, p. 18).

Importantly, especially for the connection between Camus’ absurdism and the case of AI, this problem of uncertainty in action extends beyond the practical effectiveness of our choices and into the realm of their ethical correctness. Our common moral assumptions, and even the more sophisticated moral theories, are also ultimately unfounded, tracking perhaps our cultural, personal and societal context, but without justifying the normative relevance of these somewhat arbitrary factors in turn (Camus 2013a, p. 17–18). The result is a crisis in ethics, where once we try to guide ourselves only by what we know for sure, along with what follows logically from it, we are no longer able to uphold any values, no matter whether these values are personal or cultural/societal. We cannot hold others to values and principles that we cannot justify.

And here AI as a disruptive technology forms a strong example: when AI creates for us an ethical dilemma, it confronts us, in a sense, with the unfoundedness of one or more of our ethical principles. For instance: the dilemma of accountability in AI, calling into question who is an appropriate target of responsibility and what level of moral agency we even expect from humans, reveals how Floridi and Cowls’ principle of explicability/accountability is based on yet other concepts, which are themselves underspecified for newly arising technological possibilities. We want to evaluate the new possibilities of agency dispersal and deference to technology with which we are confronted. We look within our ethical frameworks for a clear and exhaustive definition of ‘the human in the loop’, but find instead ‘silence’.^2^ It is precisely this unfounded quality of our conventionalized ethical frameworks that makes them vulnerable to technological mediation: values and principles perched atop ‘silence’ will bend not only to human need, but also to the whims of circumstance. The human encounter with disruptive technology, while being very obviously an interaction of physical entities with practical, socio-political implications, is for this very reason also a metaphysical revelation, to do with the nature of the normativity we use to navigate such situations. Thus, through an encounter with AI, we strive to ethically evaluate the technological circumstances we create, but we are confronted with the fundamental silence of our world. Therefore, we experience the absurd.

This experience of absurdity is not the end of the story, however. As both in Camus and in recent literature on the ethics of AI, we encounter multiple attempts to change our previous normative frameworks to fit the peculiar situations which we encounter through this new technology. We attempt to resolve our ethical dilemmas by introducing new concepts and by making evaluative choices. Think of Mecacci and Santoni de Sio’s claim that meaningful human control over a network depends on responsiveness to reasons, not merely actions: this is a normative decision with little argument or grounding, other than that it makes our ethical frameworks more coherent in the face of AI. It is these attempts at more fundamental normative change for the sake of moral sensibility that, in line with Camus, may be described as ‘rebellious.’ This again leaves us with a ‘deeper dilemma’, however. Because once we have undertaken the fundamental change of our framework, we have recognized its unfoundedness and rejected it on that basis. Rebellion is, in that way, a form of refusal or negation motivated by our rational drive for justification (Camus 2013b, p. 12). In other words, it is that drive asserting its own worth through refusal. How, then, when we have to reject the previous normative order, and we can no longer simply trust our (technologically mediated) judgement, can we guide and trust ourselves in building a better approach to AI? How do we ensure that ‘ethical coherence in the face of AI’ is not simply ethical subservience to the whims of technological advancement?

### AI and rebellion

The seemingly strong parallel between the human’s encounter with AI and the experience of the absurd suggests that, to reach a potential Camusian solution to our AI-ethical dilemmas, we should examine and integrate Camus’ theory of rebellious struggle for (normative) change, which he presented in 1951 in *The Rebel* (2013b). In this work, Camus explicitly addresses the question of how to go about changing our normative order while staying true to the wholehearted rejection and discreditation of the previous order. He claims we can do so by being willing to completely reconsider our societal framework of values, while still holding to two fundamental ideals: logical self-consistency and the sense of dignity which moves us towards change in the first place (Camus 2013b, p. 58). Using these in order to develop principles for guiding normative change will be of importance, because the re-evaluation and adaptation of our normative frameworks is necessitated (and simultaneously mediated) not just by artificial intelligence but also by any new and impactful technology. We thus have to recognize the need to continuously re-evaluate our ethics in response to novel developments, and the need for guidance in such upheavals. Camus himself underscores this when he reflects on the technological advancements of his own time, in The Rebel (2013b). According to him, industrial technology, which increasingly had taken over human tasks and alienated human labor from its end-product, “already demonstrates, by the way that it functions, the necessity for moderation and gives rise to reflections on the proper way to organize this moderation” (2013b, p. 237). AI is only an especially good impetus for this same type of recognition, since its disruption of human practices and industries calls into question many ethical values and principles at once, which currently form the fundament of our society: i.e. privacy, autonomy, accountability, justice/nondiscrimination, etc. It provides us with a particularly intense experience of the absurd, standing in doubt about how to fundamentally redefine a large portion of our collective normative conventions. AI, in this way, is a problem which motivates a multi-purpose solution: a solution to its own—as well as other problems of the same sort. This solution, again, is a guidance of fundamental normative change by ideals of rebellious self-consistency and human dignity.

Camus, in *The Rebel*, analyzes the concept of ‘rebellion’ as a form of action or choice, which has both a positive and a negative content (2013b, p. 1). On the one hand, rebellion is a form of refusal, refusing to accept a certain state of affairs and therefore a demand that it should change. On the other hand, rebellion is a refusal *in defense of something*. That is, if a certain state of affairs is unacceptable to a person, then it is unacceptable in light of a particular value (Camus 2013b, p. 1–2). Additionally, it is possible, perhaps even inevitable, that rebellion against a particular state of affairs calls into question not just some standing material situation, but also the normative order which underlies (and justifies) it (Camus 2013b, p. 33). This can be seen most fundamentally in what Camus calls “metaphysical rebellion”, in which the human comes face to face with the absurdity of their own existence and rebels against it (2013b, p. 12). Seeing how the natural order- or ‘facts’ of the world deny the human any certain or stable sense of meaning, or of normative guidance—as we previously discussed—the human can respond by rebelling against this world (or reality, or nature). They thereby *refuse* to accept any normative order based (solely) in the (natural) facts of this world (in other words, a value-system based on arbitrary circumstance).^3^ This refusal implies a full break with any conventionalized normative order, since these tend to assume that certain basic values or normative principles are simply matters of fact in the rational order of reality (or, at least, they take certain values and principles ‘for granted’) (2013b, p. 35–36).

Camus calls this overthrowing of the conventionalized order “metaphysical revolution”, and sees as the prime example the total destruction of God as a source of meaning or values (2013b, p. 35). Yet, whether this overthrowal of the standing normative order is religious or secular, a break with trust in God- or a break with trust in the natural world to ground our normative sense, this metaphysical revolution is the conceptual basis for every refusal and overthrowal of a normative order, from the practical-ethical to the political. It is worth remarking here that Camus does distinguish between the aforementioned “metaphysical rebellion/revolution” and its political, historical counterpart. While they share an origin in one’s experience of the absurd and a fundamental grounding in human dignity, the two concepts have a different function. Metaphysical rebellion serves to help us think about refusing and changing those intangible normative frameworks by which we understand ourselves, and by which we structure and approach the practical situations which shape our lives (Camus 2013b, p. 13). Historical rebellion is more concerned with the tangible, embodied normative frameworks (and their absurdity), such as systemic injustice, tyranny, and slavery, and how violence may or must be employed to change them (Camus 2013b, p. 58).

Though AI itself is a very practical reality, ingrained in a political context and physically incarnated rather than metaphysical, the human’s encounter with it and the absurdity thus experienced can be of either a metaphysical or social/political nature. Or, what is more likely, a combination of the two kinds of absurdity is experienced and leads to a similarly multifaceted rebellion. Encountering AI-based decision-making and societal structure can obviously lead to political, historical rebellion wherein we question and reject the standing political, corporate or social policies which we find lack justification, along with the laws and leaders that uphold them. However, we may also, simultaneously or subsequently, question the very basic normative assumptions and frameworks on which we base our lives. In this case, we would especially question the normative status quo around our relation to technology generally speaking, since this status quo does not provide us with satisfying answers to new technological dilemma’s. In fact, the status quo framework of ethics is mediated by the very technologies that face us with situations that seem unreasonable and unjust to us, wherefore we must mistrust and reject even this intangible status quo itself. It is this step, with which this article, focusing on ethical deliberation and reconsideration in the face of concrete dilemmas (as opposed to political policy-change or activism), is primarily concerned. This is, quite clearly, primarily a matter of metaphysical rebellion and revolution, wherein “the rebel realizes that it is now his own responsibility to create the justice, order, and unity that he sought in vain within his own condition” (Camus 2013b, p. 13). The reappraisal of ethics discussed here is also far from immaterial to addressing the more tangible, political, and historical absurdities of a society disrupted and mediated by AI, as reconsidering our general and situational ethical frameworks around technology will inevitably feed into policy changes or activist agendas.

However, this task of rejecting and rebuilding our normative frameworks leaves us with an important question: if we reject the current ethics (and other standards), then based on what value or principle will the whole framework be changed (Camus 2013b, 36)? Here we see again the clear parallel with the case of AI and the general adaptation of our ethical frameworks: we find that the current frameworks are not sufficient or acceptable, we demand that they change, but because these frameworks which make sense of our values are themselves untrustworthy, we seem to have no values/principles based on which to guide our changes.

The solution, for Camus’ rebel as well as for us in the case of disruptive technology, is that we must guide ourselves by the very idea, value or concept which animated our call for change in the first place: “The consequence of rebellion […] is to refuse to legitimize murder because rebellion, in principle, is a protest against death” (2013b, p. 227). Here the fundamental practical logic of rebellion comes to the fore; namely that because all transformative/revolutionary action is undertaken with an underlying defense of a value as its object and motivator, any way of conducting that action which contravenes or disrespects that same value is (logically and evaluatively) inconsistent with itself. This is what we shall, in this article, call the ‘logic of rebellious self-consistency’: a particular sense of self-consistency which is internal to rebellion, ensuring its evaluative perspective and its practices do not become self-contradictory.

It must be noted here that although in rebellion and revolution we cast off our previous normative order, this does not mean that we are not still motivated ethically towards change. The operative term in our ‘normative order’ being ‘rejected’ through rebellion is ‘order’, as opposed to ‘normative’: i.e., we are not abandoning normativity or discrediting any particular ethical concepts, such as privacy or justice. Rather, we reject the way these were previously structured and justified in a ‘normative order’ or ‘framework’. This is how, for Camus, there can still remain a fundamental intuition of a particular value which motivates us towards rebellion, even if no value *framework* is recognized any longer in the course of this movement. The ‘revolutionary’ nature of this value, which motivates and guides the coming changes, is then also not in its conceptual uniqueness (inventing a totally new value or concept) but rather its solitary nature (there will be no other values for this intuitive sense of value to be traded off against).

For the metaphysical rebel, for anyone who fundamentally questions the normative order of things, this underlying value can only be that of human dignity. After all, metaphysical rebellion and revolution, while they may be inspired by concrete social, political, or historical events, are most fundamentally aimed at the absurdity which is encountered in those situations: they are aimed at the unreasonable nature of human existence/reality, whereby the rational drive for understanding, and action-guidance by well-founded principles, is frustrated (Camus 2013b, p. 12). If the objectionable feature of reality and the standing order of things (be it in morals, politics or science) is that it does not satisfy this drive to understanding, certainty, and ‘decency’, then naturally the fundamental motivation for rebelling against said order must be the perceived value of that same drive (Camus 2013a, p. 39–40). The human who rebels here is, then, a self-perceived rational agent who acts for the sake of rational agency and its inherent drives in and of themselves. It follows that they act in protection of their own self, or their own humanity, as they conceive of it, that is: they rebel in defense of their inherent value or ‘dignity’ as a rationally acting being (Camus 2013b, p. 3). Put differently, rebellious ‘dignity’ is the (self-)perceived moral status of a being frustrated in a reflective, rational, evaluative way, defined in terms of their capacity for that very kind of frustration. And, since this value or ‘dignity’ is based on a faculty which we can recognize in others as well as ourselves, Camus’ rebel acts of necessity for the sake of human dignity more generally, i.e. for the dignity of themselves and others in equal measure (Camus 2013b, p. 9–10). Finally, the reason this sense of dignity holds up for the rebel despite the fact that they have called into question/rejected any normative order which surrounds them, is that this sense of worth is the necessary condition for the act of questioning/rejection itself. The aforementioned logic of self-consistency demands, in fact, that the rebel sticks by their, and others’ dignity.

The reason our normative frameworks, values, and principles were called into question was that they could not find grounding in the empirical world, i.e. in our direct experience. For instance, we can see in technological mediation that material, observable conditions may *explain* the way our normative frameworks are shaped but cannot justify them. Yet, ‘dignity’ in Camus’ sense does not depend on any fact other than that we experience ourselves as rationally, evaluatively acting, that this action is frustrated by a *lack* of (empirical) grounding, and that we then choose to insist upon our reasoned action regardless: “I continue to believe that this world has no ultimate meaning. But I know that something in it has a meaning and that is man, because he is the only creature to insist on having one” (Camus 1961, p. 29). In short, the problem of grounding values and principles on concepts taken ‘for granted’, subject to ‘the whims of circumstance’, is solved by basing a fundamental value only on first-personal, basically internal phenomenal data. This is how we avoid relying on conventionalized concepts with inexhaustive definitions which terminate in a frustrating ambiguity or ‘silence’. If you see yourself as a rational agent who is frustrated with the normative order in terms of its unreasonable nature, then on pain of practical-logical self-consistency you must respect human dignity in yourself and others, and attempt to act on it.

### The Camusian approach to technological mediation

We have thus shown how Camus provides both the impetus and the guidance for change of a given normative order. The key is that for Camus the attempt to change the order of things has its own internal logic (‘logic of rebellious self-consistency’, mainly revolving around self-consistency in motivation and evaluation) and its own internal value (‘dignity’, a value which underlies any fundamental questioning of the status quo), which together build from the individual experience of rational frustration a consciousness of common value and therefore of social, moral reasons to act: “I rebel—therefore we exist” (Camus 2013b, p. 10). The question which remains, of course, is how this helps us in the case of AI.

In the case of A.I. and other disruptive technologies, this article argues, we encounter the same fundamental rational frustration which Camus describes in ‘the absurd’, and, therefore, the concept of ‘dignity’ (and self-consistency) provides a similar solution. We are faced with realities and opportunities to which we want to adapt our behavior and our frameworks of understanding, to feel secure in the knowledge that we act responsibly. Yet the very nature of these technologies and what they do to influence our context of living prevents us from ever truly knowing whether our choices are optimal, victimless, proportional, or fair. The ‘silence of the world’ against which Camus describes the human being to stand here can be likened quite aptly to the person who is presented with a credit score generated by a deep-learning AI which does not fully justify its decision, or the AI-ethicist who looks into their own values and intuitions and cannot tell exactly how and where they may be biased by technological mediation (2013a, p. 22). In any case, reality frustrates the rational drive, the drive for ‘decency’, for knowing exactly what we are doing and why, because we cannot find in reality a justification for certainty (Camus 2013a, p. 17). We similarly want to change either the realities we face, i.e. the technologies, or the concepts and principles we use to understand and act around them, i.e. adapting our normative frameworks. And we similarly run into indecisiveness when we then inevitably find that we cannot fully trust any of our standing normative values and principles. Yet, by applying the logic of rebellious self-consistency in motivation and the concept of common dignity, we can start to see particular directives for our changes in normative framework and technology. The common dignity which motivates our normative rebellion and revolution in the first place can remain centered only if we codify it into overarching principles which are, in the course of normative change, supreme.

This may seem like a counterintuitive proposal, considering that Camus’ philosophy is also heavily concerned with the dangers of ostensible reason and virtue gaining a totalitarian kind of sway in our inner and our political world (2013b, p. 83). For Camus, an abstract normative value or principle should never become disconnected from and more important than the actual people (i.e. the general human dignity of real humans) it was invented to protect and advocate for: “Virtue cannot separate itself from reality without becoming a principle of evil” (2013b, p. 238). Yet, at the same time, we have seen through Camus that a normativity based entirely on our current experiences and understandings of the real world is always inherently compromised, because it is liable to change itself based on the whims of circumstance. Our ethics cannot “identify itself completely with reality without denying itself” because doing so would turn it into blind assent to the status quo (2013b, p. 238). The only other option, then, seems to be for ethics to be in a constant tension with reality, not to divorce from or dominate it but to simply seek to change it: “The procedure of beauty, which is to resist the real while conferring unity upon it, is also the procedure of rebellion” (Camus 2013b, p. 220). What must be protected by all means, then, is the condition of normative change, of continual upheaval or the potential for it. And this, as even Camus attests, must happen through the values and principles internal to rebellion, which are also translatable into and guarded by certain baseline rights or preconditions:

A revolutionary action which wishes to be coherent in terms of its origins should be embodied in an active consent to the relative. […] it should demand absolute freedom of speech. Thus it would preserve the common existence which justifies its insurrection. In particular, it should preserve, as an absolute right, the permanent possibility to express one’s thoughts. […] There is no justice in society without natural or civil rights as its basis. (2013b, p. 232)

The leap, then, from what Camus explicitly attests and proves necessary to the proposal of a framework of meta-level principles for ethical deliberation and change, is not so far as it may at first seem. That is, so long as we keep in mind that these meta-level principles are not formulae for deducing the right ethical approach in any concrete situation. They are merely guidelines by which to structure our deliberation on ethics in a situation. According to Camus, “[r]ebellion, in itself, is not an element of civilization. But it is a preliminary to all civilization" (2013b, p. 216–217). This means that even if rebellious morals and strategies cannot replace our everyday normative frameworks, the principles implied by rebellion are necessary guidelines to those frameworks’ proper (re)construction, as part of a constant state of flux (a state of change or potential for change). And so it is necessary to a just normative framework that we are also able to formulate and follow at all times these meta-level principles of ethical deliberation.

### Three Camusian meta-level principles

This final section seeks to establish that if we undertake changes in our normative, ethical frameworks for AI and other technoscientific innovations, then these changes must adhere to at least the conditions of inclusivity, democracy, and self-limitation. These meta-level principles are inferred from the foregoing Camusian analysis of rebellion and the absurd, most importantly from the value of human dignity and the logic of self-consistency which are internal to rebellion. These principles are all, in a sense, different formalized expressions of human dignity and the importance of self-consistency, and so they will naturally lead into one another. The last of these principles is likely the most unconventional, and it is also the broadest and most abstract, so we shall begin by explaining the condition of self-limitation.

#### Self-limitation

Within rebellion there are at least two fundamental presumptions: the status quo is unjust (or in need of change), and it is unjust in relation to rational, evaluative agents who are of inherent value (Camus 2013b, p. 59–60). This means that, because the initial state of matters is unjust, the attempt to effect meaningful change is always going to be a struggle: if the standing order of things was perfectly responsive to the need for change based on the dignity of its subjects, then rebellion would not have been prompted. Therefore, attempts to change will face resistance, be it deliberate or not, and be it from the agency of other people or from circumstantial factors. Camus himself recognized this aspect of rebellion when he noted that “[if] rebellion exists, it is because falsehood, injustice and violence are part of the rebel’s condition” (2013b, p. 227). The change intended by rebellious action can thus turn out imperfect or fail to materialize. This in turn motivates instrumentalist reasoning: if resistance is expected, and failure is possible in various measures, then we are naturally pragmatically incentivized to use any means which contribute to expedience, to maximizing the likelihood and extent of success. Even if instrumentalism is not the preferred method of the rebel, they cannot eschew it entirely in an imperfect world, without surrendering in some sense their commitment to the dignity which they claim to defend. If human dignity is truly of supreme value, then we must, after all, maximally defend it. As Camus himself puts it, a rebel cannot “absolutely claim not to kill or lie, without renouncing his rebellion and accepting, once and for all, evil and murder” (2013b, p. 227). In a broader sense, this represents Camus’ admission that in an imperfect, unjust world it is impossible to change anything for the better without employing means of expedience which create friction, (temporary) polarization, and at times even more direct, material harm. Even specifically in the context of technological advancement, Camus seems to emphasize this:

The very forces of matter, in their blind advance, impose their own limits. […] The machine is only bad in the way that it is now employed. Its benefits must be accepted even if its ravages are rejected. (2013b, p. 237)

This makes the instrumentalist reasoning under discussion necessarily a calculation of harms against benefits. A very simple example in the ethics of AI is that of *when* we decide on a change (in norms, guidelines, or even legislation): deliberating longer may improve our normative frameworks, and holding off from innovating until that time may prevent additional unintended harms, yet ‘waiting’ like this also risks the longer persistence of current harms, as well as missing out on the benefits of technological innovations for longer. A choice in this regard is always an imperfect balance of real, material harms and benefits.

However, the fact that the world is unjust in relation to the inherent value of rational agents also means that rebellion is always enacted fundamentally for the sake of everyone else, as well as oneself, as beings of equivalent moral value (Camus 2013b, p. 3). This means that any harm or disrespect that becomes the dignity of one person, can only be compensated with the dignity of another. If not, this compromises the very core of the rebellion, since it is now prioritizing abstract ‘results’ over actual people. This principle is similar to the rationale in human rights theory that one right, or one person’s right, can only ever be overruled through the competing right of another (Hart 1955, p. 178). Taken in combination with the previous notion of ‘imperfect’ choices, this principle implies a stringent limitation on how imperfect our choices are allowed to be. An attempt to better the world can only go so far in its instrumentalist reasoning as to not disrespect the dignity of individuals, especially without direct compensation in kind. For example: Camus was famously opposed to the more authoritarian incarnations of socialism because, as he claimed, they traded the freedoms of people now for the sake of the prosperity of a nonspecific group of beneficiaries in the distant future (Foley 2008, p. 141). For Camus, as echoed by John Foley, the ends cannot always justify the means, because “when we live towards ends, the bodies pile up” (2008, p. 124). In other words, if an end justifies *any* means necessary to achieve it, then this includes means which treat people as mere pawns and disrespect human dignity, which means we should re-evaluate our ends. The only supreme end is that of the dignity of tangible people: if not people that are already here with us now, then at least people we can specifically expect and envision, not any entirely hypothetical person in an idealized future. Thus, the principle of self-limitation implies that even in trying to change an unjust, imperfect society, we must accept a limit to the means we can use towards that end. This is so even when accepting a limit on our actions risks total failure. After all, within rebellious action, a success that contravenes human dignity is in truth no success at all, but rather a moment of self-defeat. It is rebellion forgetting its original impetus, a breach of the logic of self-consistency. In Camus’ words: “[…] rebellion with no other limits but historical expediency signifies unlimited slavery” (2013b, p. 236). The principle of self-limitation implies that self-imposed limits, along with the frustration and risk of failure they may bring, are at times in fact desirable, because they are just another way to express the value of human dignity:

If the limit discovered by rebellion transfigures everything; if every thought, every action which goes beyond a certain point negates itself, there is in effect a measure by which to judge events and men. […] At the same time that it suggests a nature common to all men, rebellion brings to light the measure and the limit which are the very principle of this nature. (Camus 2013b, p. 236)

‘Dignity’ here must be taken quite broadly. It can be taken to refer to the inherent value of a person as a reasoning being, implying at least the consideration of their interests and desires in equal measure to any other person, as well as their freedom to express those interests (Camus 2013b, p. 3–4). In the context of self-limitation, then, human dignity is both the goal as well as the limit on how we may go about achieving that goal.

On a meta-level, what self-limitation will imply for the concretization of abstract ethical principles of AI, as well as for the resolution of dilemmas, is a direction on the admissibility of certain reasons within ethical deliberation. As we previously saw, Camus points specifically to industrial technological advancement when he says that there is need of “moderation” in development, as well as of a “proper way to organize this moderation” (2013b, p. 237). The principle of self-limitation, or, as Camus here terms it, the “law of moderation”, provides guidance on precisely this organization, since it explicitly positions the interests of potentially affected humans, as well as the expression of such interests, as overriding reasons. Reasons of an instrumental nature cannot be used to counter reasons or principles which defend the dignity of particular, identifiable people. For instance, if a technology has as its goal the safety of people, we cannot reject the integration of principles into its design that protect the safety of particular people from its misuse, even if this would put instrumental limitations on the operation of the technology. The only exception, of course, being if an equal amount of identifiable other people would be similarly endangered by the aforementioned instrumental limitations. Actually calculating these matters may often prove impossible in practice, seeing how tallying stakeholders and their interests, as well as the degrees of each’s interest, in a certain concrete guideline would be beyond the capabilities of many forums for deliberation. Yet, this does not make the principle useless in such a scenario. Principles, after all, are useful also in cases of incomplete information, such that we at least strive to choose what is more respectful of our common dignity, instead of choosing arbitrarily, based on expediency, or based purely on intuitions which may be biased by our context. Camus himself warns of the dangers in technological advancement, which marches on entirely heedless of the human who must live and work within this artifice: “Either this value of limitation will be realized, or contemporary excesses will only find their principle and peace in universal destruction” (2013b, p. 237). To formulate the principle of self-limitation positively: if a limitation and its concomitant risks express the common dignity of everyone more than does the rejection of that limitation, then this limit ought to be imposed. This meta-level principle implies that we must be willing at times to accept the potential failure (or postponement) of a technology, for the sake of taking all interests into serious consideration. This also means accepting the limitations implied by a democratic process, which leads us into the next principle.

#### Democracy

The aforementioned need to take seriously everyone’s interests to the best of our ability leads directly into the principle of democracy. Because any person is ultimately, owing to their common human dignity, of equal moral worth to any other person, it naturally follows that any change of the (normative) order of things ought to be directed as much as possible by all potential beneficiaries of that change. This seems to follow naturally from the previous principle, and seems to be what Camus means when he says that “[…] the affirmation of a limit, a dignity, and a beauty common to all men only entails the necessity of extending this value to embrace everything and everyone and of advancing towards unity […]” (2013b, p. 196).

This principle may read as common sense, but it has the potential to be quite radical, because there is no other reason (outside the three meta-level principles) that can be levied against this demand for democracy. Efficiency, for instance, or economic rights of property, do not have an equal sway to that of democracy (or inclusivity). While Camus himself vocalized support of a “liberal politics” in his non-philosophical writings, the supremacy of equity over economic incentives should mark a clear difference between his understanding of ‘liberalism’ and the socio-economical liberalism with which we are familiar in the modern day (Foley 2008, p. 36). It is true that this principle of democracy stands in relation to a longstanding liberal political-philosophical tradition, and this does give it the deceptive sheen of a broad, abstract principle that has nothing new to add. However, on the one hand, and in line with Camus, this article argues that we must avoid introducing values and principles “foreign to history” as much as we must avoid introducing redundant ones (2013b, p. 194). On the other hand, we should emphasize that our notion of democracy is absolute, as opposed to the purely historical sense of democracy, which is at all times somewhat compromised by external factors such as bureaucracy and socio-economic power concentration. Nor is our sense of democracy an empty formalism, since it specifies that only those whose interests and dignities are at stake in a particular, concrete case of technological integration or disruption are designated to take part in the concomitant ethical reappraisal.

The more instrumental values, such as efficiency and economics, are only worth anything here to the degree that they contribute to the dignities and basic interests of (identifiable) people. For example, it is possible that, even under the principle of democracy, some people may be barred from weighing in on a particular deliberation, if having to wait for their input to make a decision could cost another, roughly equal set of people their lives. These types of considerations become relevant when ethical deliberation feeds directly into decisions about a particular technology, or into otherwise more tangible policy. This would then involve a reason of efficiency, but one which indirectly points back to human dignity in a clear and concrete way. In a sense this is also an expression of the principle of self-limitation: maximal democracy is not an end in itself, but an expression of human dignity, so the degree of democratic participation may be limited if that limitation would express the dignity of more people (or express their dignity to a greater degree). On the other hand, it seems unlikely that anyone can be denied a voice in the design and deployment of a new technology purely on the rationale that the technology is developed and owned by a particular company. This is a reason purely of property, with no clear connection to the interests and desires of more than a few people, such as shareholders and CEO’s. Given enough other societal stakeholders, and/or given that a technology would affect other societal stakeholders more than it would those who own and profit from it, the principle of democracy would clearly demand that these stakeholders be given a voice in design, development, deployment, etc. A limitation based on property would not express more respect for common dignity than its rejection, and so the principle of self-limitation does not come into play here. If this inclusion of other stakeholders is economically inconvenient, that is also by no means an argument against it. Analogous to Camus’ defense of trade unionism, involving and empowering the masses in the production of the technology that surrounds them is simply “the negation, to the benefit of reality, of bureaucratic and abstract centralism” (2013b, p. 239).

Developing a democratic mechanism for any given context would be beyond the scope of this article. For our purposes we must at least note that any particular ethical (re)consideration surrounding a novel technoscience, in a given context, must be as democratic as the context will practically allow. If we imagine a given AI tool developed and/or used for public safety by the Dutch police, we would then be inclined by the principle of democracy to have the tool’s design and implementation (including whether to create it at all) be subject to a significant process of participation with stakeholders from industry, citizenry, government as well as police. Similarly, if an AI tool should create an ethical dilemma—or other occasions for reconsideration of ethical frameworks—in another particular societal context, then the (re)construction of our ethical framework in response must also arise from some method which integrates the perspectives of all relevant stakeholder groups. Conceivably, this could come in the form of a co-creative ethical research initiative, wherein a fairly assembled set of stakeholders from different stakeholder groups is allowed to collectively define both the ethical questions being asked and the answers to them. Stakeholder engagement should, in principle, be maximized to the point of (but not beyond) diminishing returns on the dignity of those concerned. Thinking for instance of engaging private persons and vulnerable groups, it may be against their reasonable interests (i.e. their dignity) to request extensive participation without compensation, such that the amount of participation from this group may be limited by the resources available for fair compensation. This limitation on the participation of one group could, in turn, be argued to necessitate the limitation on participation of other groups (e.g. equalizing the representatives from each stakeholder group), in order to avoid imbalance in the input of different groups. But this leads us into the principle of inclusivity.

#### Inclusivity

Even when people cannot actively advocate for their own interests, rebellious change is implied by common dignity and self-consistency to be compelled towards maximal inclusivity. After all, if every human is of equal moral worth, then in principle any change to the (normative) order of things must be aimed at benefiting as many people as possible and must consider the interests of everyone equally. To diminish someone’s worthiness of moral consideration based on some additional fact is, for a rebel who has questioned everything else in common-sense morality, to undermine the absolute nature of human dignity, and, therefore, to undermine the moral status of everyone. As such a change in for instance data- or AI legislation must be couched in the normative deliberation and consent of all whose interests are at stake: not just companies, not even just the citizens of a particular legal territory, but also the residents of any other affected part of the world *and* the conceivable future generations (to the degree that their interests can be anticipated).

It is entirely conceivable that, even if handled ‘efficiently’ through representatives of stakeholder groups, this complicates matters in terms of norm-setting and decision-making. Different ‘factions’ of stakeholders will inevitably be at odds in terms of their interests and views, which slows down decisions and therefore results. It may also, if seriously applied at several levels, lead to much less uniform norms and policy across local, national and international bodies. Perhaps most fundamentally, it could lead to a state of ethical uncertainty within our cultures and subcultures, about where exactly we stand as a society in regards to technologies. We may yearn again for one abstract set of principles, vague but clean, and one set of national or international policies, compromised by assent to the status quo but madly efficient. However, in The Rebel Camus argues clearly that disagreement and uncertainty among groups is preferable over uniformity, especially when he analyses the error involved in the French Revolution and Saint-Just’s elimination of factionalism:

No one will dare to imagine that, since factions exist, the principles are conceivably wrong. Factions will be condemned as criminal because principles remain inviolable. […] When neither reason, nor the free expression of individual opinion, succeeds in systematically establishing unity it must be decided to suppress all alien elements. Thus the guillotine becomes a logician whose function is refutation. […] Factions join with factions and minorities with minorities, and in the end [Saint-Just] is not even sure that the scaffold functions in service of the will of all. (2013b, p. 77-78).

This was a historical rebellion and revolution, but the analysis also involves an error in the underlying, metaphysical rebellion: denial of factions leads to tyranny, of the majority or of the powerful, but also the fundamental denial of difference in normative perspective leads to the rule of a truly arbitrary, unaccountable set of principles. Insistence on unity and efficiency in reasoning, to the detriment of disagreement leads, then, paradoxically, to the most absurd normative frameworks. Saint-Just, Camus writes, is killed by the very state apparatus which he wielded to condemn the factions, exalting this oppressive power even as he himself has now become the minority to be eliminated (2013b, p. 79). From this we argue that the law and especially the ethical frameworks under it should be amenable to factions, to difference, and therefore pluralism and inclusivity must be adhered to as much as is possible without undermining the conditions of social interaction which enable deliberation.

Here we also stumble upon the potential solution to a previously stated problem of AI ethics: the aforementioned ‘Meaningful Human Control’ perspective on accountability in AI development, deployment, and use seemed to lack the rigorous theoretical grounding to reliably define what ‘responsiveness to *the right reasons* of relevant agents’ meant (Santoni de Sio and Mecacci 2021, p. 1078). When are accountability mechanisms supposed to spring into action, in response to what ‘wrongful reasons’? Now we can see that ‘the right reasons’, to which a ‘socio-technical system’ (with or without AI) must be responsive, are those reasons which take into account- and respect the dignity of all relevant stakeholders of the system. This means that we can build on the accountability theory of Mecacci and Santoni de Sio to concretely hold actors in AI-adopting systems accountable to respecting all stakeholders (including citizens and future generations, for instance) in their capacity as rational agents with dignity, which demands a certain freedom and facilitation of basic interests. We here again define basic interests as those which are necessary conditions for the successful operation of rational agency: so, interests which facilitate learning relevant knowledge, setting one’s own goals, and expressing one’s opinion openly. This is just one way in which the meta-level principle of inclusivity may help in concretizing abstract ethical principles such as accountability or explicability. Of course, further concretization is still necessary: what exact interests are of concern, and by what policies of a socio-technical system must they be facilitated or at least protected? But this is the exact process of ethical deliberation which the meta-level principles are intended to guide, not to solve out of hand. They provide the guidelines by which these normative choices are to be made.

The principle of inclusivity here also supplements the principle of democracy. It emphasizes that, within a democratic ethical discourse, not only must all relevant stakeholders be included, but also everyone’s input in this discourse should be given equal weight. As such, the principle of inclusivity dictates that while (re)considering our ethical frameworks, and/or specifying what we mean by abstract principles in a particular context (such as with Floridi and Cowls’ principles for AI), we must as best as possible mitigate standing relations of power and marginalization, which may prevent certain voices from being heard or equally weighted. The ideal results of these three meta-level principles are context-specific customs, systems and/or protocols for ethical (re-)evaluation in the face of novel technoscientific innovations, able to recurrently and sustainably reconsider or concretize the meaning of abstract ethical principles such as accountability/explicability within their specific societal contexts. The function of these principles, then, is indeed not to help deduce ethical principles and decisions like formulae, but rather to guide and structure normative deliberation in such a way that it becomes a sustainable, continual process. They should mitigate in us the apprehension from making ethical decisions, for fear that our judgements are technologically or otherwise mediated. After all, our ethics are less likely to simply affirm standing socio-economic relations when all humans affected get to speak with the same authority as technocratic centers of power, and we are less likely to uncritically accept the integration of technologies into our lives if all the stories about different absurd encounters and disagreements with them are amplified with the same microphone.

## Conclusion

Having established these meta-level principles is only the beginning of adapting our normative- and other societal structures to the disruptive technology of AI. However, this is a feature, not a bug. These very principles imply that, for instance, changes made in an ethical framework for AI in a particular societal context cannot be constructed by a single author, nor even by a group of academics and scientists. It essentially requires both the consideration of the interests and the inclusion of the voices of every beneficiary or stakeholder from the very outset. This may imply, practically speaking, a rather advanced form of co-creative ethical research and a co-creative development of guidelines, fostering a process of co-design for particular AI applications. It implies the need for a process that constructs the more concrete practical directives in a given context of (potential) AI-adoption, based on the interests and ethical perspectives of all stakeholders within that context. All this under the condition that the resulting system of ethics, or the AI design, does not disrespect in any way the basic dignity and moral worth of any stakeholder.

This in itself may require additional investigation into methods of democratic inclusion and deliberative democratic principles, to both ‘get everyone at the table’ and ‘allow everyone to speak’, to make everyone’s input count. At the very least, there must be established a way of creating solutions for potential AI use-cases which is satisfactory to every stakeholder *group*, to ensure that there is no distinction made in the worth of different perspectives based on types of persons, ensuring epistemic inclusion while maintaining the Camusian sense of ‘common dignity’. This article should be a first steppingstone for subsequent research into these particular avenues of ethics in AI. It sets down the basic principles based on which we can explore our academic and societal options, with the end goal of making ethical (re)consideration(s) of AI in concrete contexts a robust and consistent enterprise. Such a consistent enterprise will be necessary as, after all, the absurd condition of our simultaneous ethical doubt and innate drive towards moral sensibility requires from us a continuous rebellious resistance. It requires this, especially, as technological advancement moves ever onward, heedless of our consent, thoroughly embedding itself into our practices and perceptions. We must resist taking our values and principles for granted, and we must resist letting technological change pass without interrogation. As Camus attests, “the rebel does not deny the history which surrounds him […] But confronted with it he feels like the artist confronted with reality; he spurns it without escaping it” (2013b, p. 231). AI is a landmark case for this essentially perpetual struggle, and how we respond to it may inform much of the future for a humanity which finds itself more and more confounded by its own artifice.

## Data Availability

No datasets were generated or analysed during the current study.

## Footnotes

1 We refer to Camus’ philosophy here as ‘moral’, but throughout this article we will use the terms ‘moral’ and ‘ethical’ more or less interchangeably. If we see any difference between the two terms, it is that ‘moral’ values are specifically other-regarding and therefore social, whereas ‘ethical’ values have to do with good living more generally and are thus more encompassing. We do not foresee this making a difference in the context of the article, at any rate.

2 ‘Silence’, not in the sense that there is no debate, but ‘silence’ in the sense that there is no definitive answer.

3 This includes more relativistic bases for an existing moral order, because the metaphysical rebel has already established that no certain facts about the world (which, for Camus, are only the directly observable, empirical facts) can give justification to an ethical value or principle. As such the metaphysical rebel is beyond the relativist’s conclusion that particular ethics are valid relative to a particular personal or cultural position. Instead, they see that no fact whatever can validate an ethics.
